# Absence of cell surface expression of human ACE leads to perinatal death

**DOI:** 10.1093/hmg/ddt535

**Published:** 2013-10-24

**Authors:** Annie Michaud, K. Ravi Acharya, Geoffrey Masuyer, Nicole Quenech'du, Olivier Gribouval, Vincent Morinière, Marie-Claire Gubler, Pierre Corvol

**Affiliations:** 1 Collège de France, Center for Interdisciplinary Research in Biology (CIRB), 11 Place Marcelin Berthelot, Paris F-75005, France,; 2 INSERM U 1050, Paris F-75005, France,; 3 MEMOLIFE Laboratory of Excellence and Paris Sciences Lettres and; 4 Department of Biology and Biochemistry, University of Bath, Claverton Down, Bath BA2 7AY, UK,; 5 INSERM U983, Hôpital Necker-Enfants Malades, Université Paris Descartes, Sorbonne Paris Cité, Paris, France,; 6 AP-HP, Département de Génétique, Centre de Référence MARHEA, Hôpital Necker-Enfants Malades, Paris, France and; 7 Centre de Référence des Maladies Rénales Héréditaires de L'Enfant et de L'Adulte, Paris, France

## Abstract

Renal tubular dysgenesis (RTD) is a recessive autosomal disease characterized most often by perinatal death. It is due to the inactivation of any of the major genes of the renin-angiotensin system (RAS), one of which is the angiotensin I-converting enzyme (ACE). ACE is present as a tissue-bound enzyme and circulates in plasma after its solubilization. In this report, we present the effect of different ACE mutations associated with RTD on ACE intracellular trafficking, secretion and enzymatic activity. One truncated mutant, R762X, responsible for neonatal death was found to be an enzymatically active, secreted form, not inserted in the plasma membrane. In contrast, another mutant, R1180P, was compatible with life after transient neonatal renal insufficiency. This mutant was located at the plasma membrane and rapidly secreted. These results highlight the importance of tissue-bound ACE versus circulating ACE and show that the total absence of cell surface expression of ACE is incompatible with life. In addition, two missense mutants (W594R and R828H) and two truncated mutants (Q1136X and G1145AX) were also studied. These mutants were neither inserted in the plasma membrane nor secreted. Finally, the structural implications of these ACE mutations were examined by molecular modelling, which suggested some important structural alterations such as disruption of intra-molecular non-covalent interactions (e.g. salt bridges).

## INTRODUCTION

Angiotensin I-converting enzyme (ACE; peptidyl-dipeptidase A) is a metallopeptidase which belongs to the gluzincin family of metalloproteases. ACE cleaves the C-terminal His-Leu dipeptide from angiotensin I (Ang I) to produce the potent vasopressor octapeptide, Ang II. It also inactivates bradykinin (BK) by the sequential removal of two C-terminal dipeptides. ACE is implicated in blood pressure regulation, water and salt metabolism, cardiac and renal function and in other non-traditional biological functions, as reviewed recently in depth by Bernstein *et al*. (1). ACE is a target for inhibiting the renin-angiotensin system (RAS). ACE inhibitors have shown their efficacy in the treatment of hypertension, congestive heart failure and diabetic nephropathy and are able to prevent cardiovascular events in patients at risk.

ACE is a type 1 ectoenzyme anchored to the plasma membrane with the bulk of its mass exposed at the extracellular surface of the cell. Two forms of ACE exist in humans: a somatic ACE of around 170 kDa found in endothelial, epithelial and neuronal cells and a smaller testicular isoform (around 100 kDa) present in germinal cells. Both forms are transcribed from a single ACE gene which results from the tandem duplication of an ancestral gene. Somatic and germinal ACE species are generated by the initiation of transcription from alternative start sites under the control of two separate promoters. Somatic ACE is involved in cardiovascular regulation and germinal ACE in male reproduction.

Somatic ACE comprises two highly homologous domains called N- and C-domains, each of which contains a catalytic site with the His-Glu-Met-Gly-His consensus sequence for zinc binding, whereas germinal ACE comprises a single catalytically active domain identical to the C-domain. Both domains are connected by a linker region of 14 amino acids. The two domains of somatic ACE exhibit somewhat different enzymatic properties: the C-domain is more effective for converting Ang I into Ang II (2). Both domains cleave bradykinin with the same efficiency (3). The N-domain cleaves much more efficiently the physiological substrate, the tetrapeptide acetyl-Ser-Asp-Lys-Pro (Ac-SDKP) (4).

ACE is anchored in the plasma membrane via a hydrophobic sequence followed by a short intracellular (ic) C-terminal tail. The C-domain of ACE is followed by a stalk region of ∼32 amino acids which is cleaved by a still unidentified ACE sheddase that is inhibitable by metal ion chelators, compound 3 (5) and TAPI-1 (TNF-α protease inhibitor-1) (6). The site of cleavage of human somatic ACE has been identified between Arg1203 and Ser1204 (7) but other cleavage sites have also been described (8) and the precise sequence in the stalk region appears not to be critical for solubilization.

The three-dimensional (3D) structures of different forms of ACE have been elucidated by X-ray crystallography: drosophila ACE which bears a single domain (9,10), the human ACE N-domain (11) and the human C-domain (human testis ACE) (12).The structure of human full-length somatic ACE is not yet available but molecular modelling based on preliminary electron microscopic data of porcine somatic ACE has reported a plausible orientation of the two domains (13).

The level of human circulating and tissue ACE is in part determined by the I/D ACE gene polymorphism (14). Patients carrying the I allele in the homozygous state have half the ACE plasma level of patients with the DD genotype. In humans, few ACE mutants have been described: (i) heterozygous missense mutations of ACE in the stalk region (P1199L) (15) or a truncated form of ACE (W1197X) lacking the transmembrane anchor (16), both leading to a marked increase in circulating ACE but without a clinical phenotype; (ii) a heterozygous missense mutation (Y465D) distal to the stalk region resulting in a marked increase in the rate of ACE shedding and associated with significant local conformational changes of ACE (17), but again, without clinical abnormalities.

In mice, total ACE gene inactivation is compatible with life, whereas in humans it leads to severe hypotension, renal hypoperfusion and renal tubular dysgenesis (RTD), a dramatic kidney disease marked by anuria or severe renal insufficiency at birth, absence of renal proximal tubule differentiation and, most often, perinatal death (18). In some cases, renal insufficiency requires chronic peritoneal dialysis from birth, as reported in the case of the missense mutant Q1069R which resulted in a total lack of ACE activity (19,20) and of the deletion mutant Δ1141–1152 (21).

Mutations of the RAS found in RTD offer a unique opportunity to analyse the structure–function relationships of the RAS proteins, as we have reported in the case of different renin mutations (22). Gribouval *et al*. (23) have reported 33 ACE mutations responsible for RTD in 31 families. In the present study, two of these mutants allowed us to decipher the role of tissue-bound ACE versus secreted and circulating ACE. The R762X mutant was enzymatically active, not inserted in the plasma membrane, secreted and the mutation resulted in fatal RTD. The R1180P mutant was also active but inserted into the plasma membrane from which it was quickly solubilized. The patient suffered from transient RTD and was a compound heterozygote with a truncated mutation in the other allele (K572NfsX40). Four other mutants were also investigated: two missense mutants, one located in the N-domain close to the interconnecting region (W594R) and the other in the C-domain (R828H), and two truncated mutants in the C-domain where the mutations were close to the stalk region (Q1136X and G1145AX).

All mutants were expressed in Chinese Hamster Ovary (CHO) and in human embryonic kidney (HEK) cells, and their properties compared with those of wild-type (WT) ACE. Using a molecular modelling approach (based on N- and C-domain structures of ACE), we were able to predict the structural alterations responsible for the loss of function of the mutants studied. Taken together, the results obtained show that the presence of membrane anchored ACE (tissue ACE) is essential for early renal development. The total lack of cell surface expression of ACE leads to RTD and perinatal death in humans.

## RESULTS

### ACE mutations in patients affected with RTD

The seven different ACE mutants responsible for RTD have been reported by Gribouval *et al*. (23). All patients had severe oligohydramnios and died within a few hours or days after birth, with the noticeable exception of one patient. Nine of these patients were homozygous for the ACE mutation and three were compound heterozygotes. The seven mutants studied corresponded to five distinct families numbered according to Gribouval *et al*. (23): (i) family DYS66: this person (thereafter referred as patient I) is the only patient of this series who survived after birth. The patient presented with an oligohydramnios and severe renal insufficiency at birth (plasma creatinine of 370 mmol/l). He spontaneously recovered renal function and his plasma creatinine was within the normal range 3 weeks after birth (45 mmol/l). However, the kidneys were hyperechogenic and remained so at 8 years of age. He is a compound heterozygote with one allele carrying a premature stop codon located after the catalytic N-domain (K572NfsX40) and a missense mutation on the other allele (R1180P). The father and the mother of the patient were genotyped (Table 1). Clinical investigation of this patient at 10 years of age and of his parents, in the Clinical Research Center of Hôpital Georges Pompidou, showed that he had a normal renal function with a low systolic and diastolic blood pressure. Table 1 shows the genotype of the patient's parents and the results of measurement of the plasma RAS components. There was a massive increase in the patient plasma renin activity (PRA) and a marked decrease in his angiotensinogen concentration, which was 10–20 times lower than that of his parents and controls, respectively. Plasma ACE activity, using Ang I as a substrate, was within the normal range. (ii) Family DYS183 is a homozygous missense mutation within the N-domain (W594R); (iii) family DYS103: a homozygous truncating mutation (R762X); (iv) family DYS51: compound heterozygotes with a missense mutation (R828H) and a truncation (Q1136X). Both mutants were studied *in vitro*; (v) families DYS204, DYS202, DYS194 and DYS209: homozygous truncating mutations (G1145AfsX12 named after G1145AX).

**Table 1.** Analysis of the plasma RAS in family DYS66

	ACE genotype	Angiotensinogen (ng Ang I/ml)^a^	PRA (ng Ang I/ml/h)^b^	ACE activity (fmole Ang II/min/ml)^c^
Patient	R1180P/K572NX	51	19.8	353
Mother	R1180P/WT	620	0.8	678
Father	K572NX/WT	661	1.9	327
Normal values	WT/WT	1 064 ± 223^d^	1.3 ± 0.6^d^	335 ± 83^e^

^a^Angiotensinogen concentration expressed as ng of Ang I generated/ml of plasma.

^b^PRA, plasma renin activity.

^c^Ang I was used as a substrate. Activity is expressed as fmole of Ang II/min/ml; normal values are expressed as mean ± SD.

^d^Plouin *et al*. (24).

^e^Nussberger *et al*. (25).

**Figure 1.**
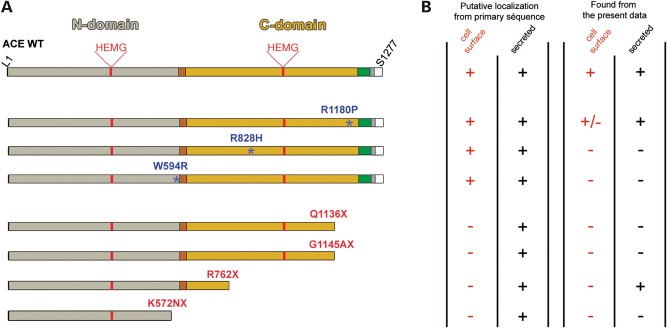
(**A**) Schematic diagram of ACE WT and RTD mutants. The ACE N-domain (L1-P602) is shown in grey, the linker region (P602-D612) in brown, the C-domain (L613-P1193) in yellow, the stalk region (Q1194-R1227) in green, the transmembrane segment (V1228-S1248) in grey, and the ic domain (Q1249-S1277) in white. Amino acids corresponding to the catalytic sites of N- and C-domains are H361–H365 and H959–H963, respectively. The positions of disease-causing missense mutations are shown by blue asterisks. The truncated mutations are indicated in red. (**B**) On the left, the table summarizes the putative cellular localization of ACE mutants according to their primary sequence and on the right, the actual localization of mutants found in cell culture in this study.

All the above seven ACE mutants were stably expressed in CHO cells for biochemical studies and transiently expressed in HEK cells for monitoring ic trafficking by immunofluorescence (IF). Their properties were compared with those of WT ACE under identical conditions. The position of these mutations in a schematic structure of ACE, the putative consequences in cellular ACE localization and its actual localization are shown in Figure 1.

### Expression of WT ACE and ACE mutants in CHO cells

**Figure 2.**
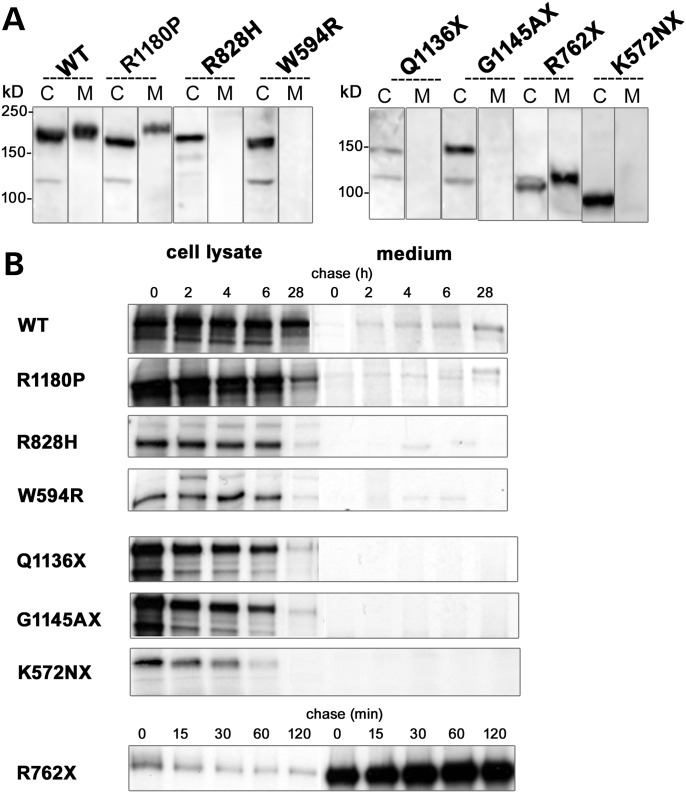
Expression of WT ACE and RTD mutants in stably transfected Chinese Hamster Ovary (CHO) cells. (**A**) Immunoblot analysis: cell lysates (C) and culture medium (M) collected 24 h after a switch to serum-free medium were analysed by immuno-blot with the ACE Ab Y1. (**B**) Pulse-chase analysis: CHO cells were labelled for 30 min and subjected to a 2–28 h chase period for WT and all mutants, except the R762X mutant which was chased from 15 to 120 min. Proteins were immunoprecipitated from cell lysates and culture medium with the ACE Ab HKCE.

WT ACE was expressed in CHO cells and found both in cell lysate and in the culture medium (Fig. 2A). Only mutants R762X and R1180P were secreted and found in the culture medium. All the other mutants were produced and found in cell lysates but were not secreted (Fig. 2A). In cell lysates, WT ACE was detected with apparent molecular weights (MW) of 182 kDa. R1180P, R828H and W594R had apparent MWs of 170, 170 and 164 kDa, respectively. The apparent MWs of the truncated mutants were 90, 113, 157 and 159 kDs for K572NX, R762X, Q1136X and G1145AX, respectively. The relatively higher MW of secreted WT ACE and R1180P compared with their ic counterparts, despite the fact that they lack the membrane and cytoplasmic domains, is likely due to their extensive glycosylation compared with the incomplete or total lack of glycosylation of the ic molecules.

Pulse-chase experiments conducted up to 28 h showed that WT ACE and the R1180P mutant appeared in the culture medium after a few hours of metabolic labelling (Fig. 2B). The truncated R762X mutant was secreted very rapidly. All other mutants were not secreted and the K572NX was quickly degraded. These results show an abnormal cellular trafficking and sorting of K572NX, Q1136X, G1145AX, R828H and W594R mutants, probably as a result of incorrect protein folding.

### Glycosylation states

**Figure 3.**
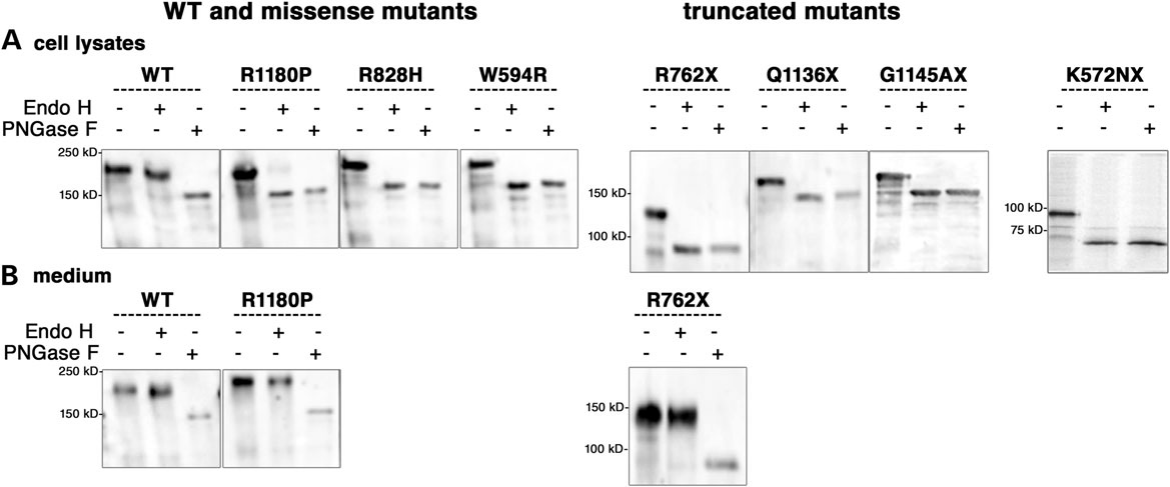
Glycosylation of WT ACE and RTD mutants in stably transfected CHO cells. Proteins were immunoprecipitated with the anti-ACE Ab HKCE from cell lysates (**A**) and culture medium (**B**). The samples were treated with buffer alone, PNGase F or Endo H and analysed by immunoblotting with the anti-ACE Ab Y1.

ACE contains 15 glycosylated sites which may be involved in the correct folding and sorting of the protein. To characterize the glycosylation state of WT ACE and the mutants, we analysed ACE in cell lysates and culture medium after treatment with either PNGase F (peptide: N-glycosidase F) or Endo H (endoglycosidase H). Endo H cleaves core-glycosylated proteins found in the endoplasmic reticulum (ER) but proteins which harbour a mature, complex glycan structure after their passage in the Golgi apparatus are insensitive to Endo H. WT ACE in both the cell lysate and the culture medium was sensitive to PNGase F and resistant to Endo H treatment, showing a fully glycosylated state (Fig. 3A and B). In cell lysate, only a small amount of R1180P mutant was resistant to Endo H treatment and showed a fully glycosylated state (Fig. 3A). All other mutants were incompletely glycosylated as they were susceptible to digestion by both PNGase F and Endo H (Fig. 3A). However, the two secreted ACE mutants (R762X and R1180P) appeared fully glycosylated in the culture medium as was the WT ACE (Fig. 3B). These data suggest that all the mutants, except R1180P and R762X found in the culture medium, were incompletely glycosylated and retained in the ER.

### ic distribution and trafficking

The ic distribution and trafficking of WT ACE and of the different mutants were studied in HEK and in CHO cells. IF studies were performed in HEK cells, using antibodies (Abs) directed against human proteins from the different cellular compartments likely to associate with ACE and its mutants. Results obtained by IF were further documented in CHO cells by co-immunoprecipitations (IP) experiments. After the removal of the final glucose residue from N-linked glycans, an essential requirement before glycoproteins can exit the ER and progress to the Golgi, ER proteins become the substrate for a chaperone family of lectins, such as calnexin (CNX). This chaperone family retains non-native protein conformations within the ER, either transiently during folding or for prolonged periods in the case of misfolded proteins.

**Figure 4.**
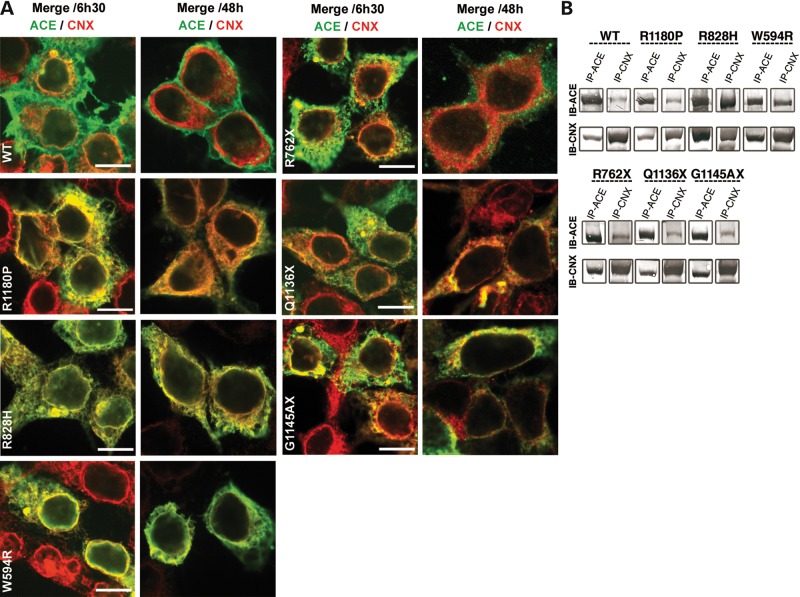
Interaction of WT ACE and RTD mutants with CNX in human embryonic kidney (HEK) and CHO cells. (**A**) Confocal analysis in HEK cells: HEK cells, transiently expressing WT and the six different mutants, were fixed 6 h 30 min and 48 h after transfection, permeabilized and stained with the HKCE Ab (green) and a CNX Ab (red). Only merge images are shown. Scale bar 10 µm. (**B**) Co-immunoprecipitations (IP) of WT and RTD mutants with CNX in CHO cells. IP of cell lysates was performed by incubation with anti-ACE Ab HKCE (IP-ACE) or anti-CNX Ab (IP-CNX). Immune complexes were precipitated with Protein G-Sepharose and analysed by immuno-blot (IB) with anti-ACE Ab Y1 (IB ACE) or anti-CNX Ab (IB-CNX).

**Figure 5.**
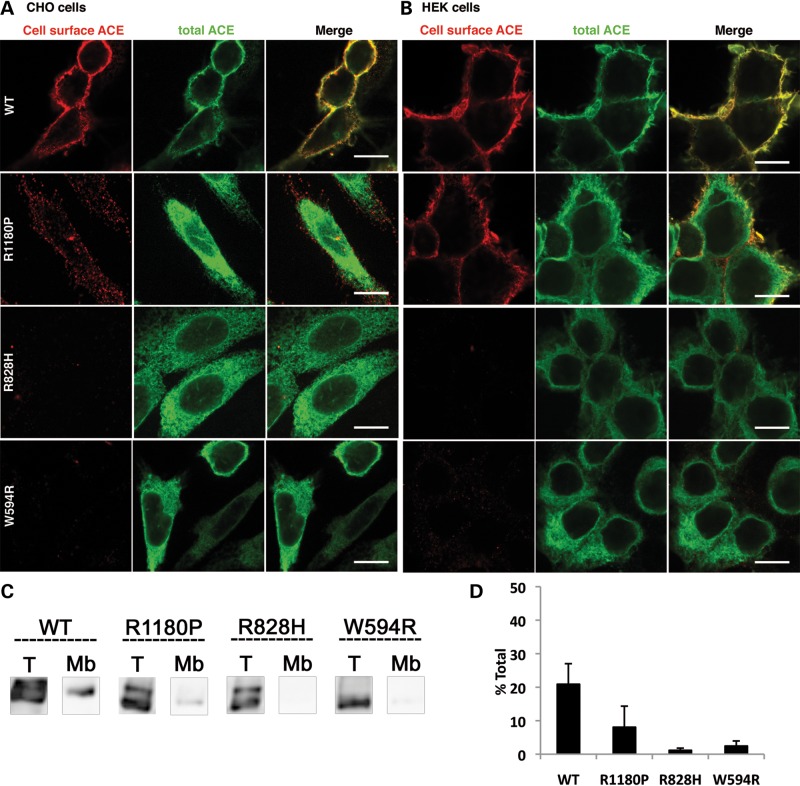
Cell surface expression of WT ACE and RTD missense mutants. WT ACE, R1180P, R828H and W594R mutants were stably and transiently expressed in CHO (**A**, **C** and **D**) and HEK cells (**B**), respectively. (A and B) IF analysis. CHO cells (A) and HEK cells (B) were grown on glass coverslips, fixed and stained with the rabbit anti-ACE Ab Y1 shown in red (cell-surface ACE). Cells were then permeabilized and the total ACE pool was stained with the sheep anti-ACE Ab HKCE shown in green. Scale bar 10 µm. (C and D) Detection of ACE surface expression. (C) Stably transfected CHO cells grown in 6-well cell culture plates, were fixed with 0.4% PFA and then incubated for 1 h with the anti-ACE Ab HKCE, solubilized and immunoprecipitated to obtain expression at the cell membrane (Mb). Total amounts (T) were obtained with cells which were fixed, solubilized and then immunoprecipitated with the HKCE Ab. Proteins were analysed by IB with the anti-ACE Ab Y1. (D) ACE protein bands were quantified by densitometry and expressed as percentages of the total ACE amount. Results shown are mean ± SD of four experiments.

(1) WT ACE was weakly associated with CNX at 6 h 30 min but no further association was detected at 48 h. In contrast, there was a strong colocalization of R1180P, R828H and W594R mutants with CNX at 6 h 30 min (Fig. 4A). The CNX pattern of these mutants was different from that of WT ACE, probably because a blockade or an accumulation of these mutants occurred in the ER. At 48 h, only the R118OP mutant appeared to have completed its cycle with CNX (Fig. 4A). There was a weak colocalization of the three truncated mutants R762X, Q1136X and G1145AX at 6 h 30 min with CNX which persisted at 48 h, with the exception of R762X (Fig. 4A). These results were further documented in CHO cells by co-IP of WT ACE and of the different mutants with CNX. Analysis of western blot data showed a slight co-IP of WT ACE and R1180P and a marked co-IP of R828H and W594R mutants with CNX (Fig. 4B). WT ACE was not retained in the ER, whereas all mutants tested, except R762X, were retained within this compartment, as found by their interaction with protein disulphide isomerase (PDI; data not shown).

(2) WT ACE was colocalized with the Golgi marker Giantin at 6 h 30 min and released from the Golgi at 48 h. The mutants R1180P, R828H and W594R were also localized in the Golgi at 6 h 30 min but their release at 48 h was slightly delayed compared with WT ACE, with the exception of R1180P which was released at the same rate as WT ACE. The mutants R762X, G1145AX and Q1136X could be detected in the Golgi (Supplementary Material, Fig. S1).

(3) WT ACE was clearly localized at the plasma membrane of CHO cells (Fig. 5A) and HEK cells (Fig. 5B). ACE expression at the plasma membrane was further documented and quantified by IP of membrane-bound ACE (Fig. 5C and D). Among all the missense ACE mutants, only R1180P could be detected by IF, although only slightly, at the cell surface of CHO cells (Fig. 5A) and HEK cells (Fig. 5B). R828H and W594R were undetectable at the plasma membrane (Fig. 5A and B). The cell surface expression of R1180P was confirmed by IP of the membrane-bound mutant (Fig. 5C and D). 10% of the total cellular R1180P was present at the membrane (Fig. 5D).

(4) The R828H and W594R mutants and the two truncated mutants G1145AX and Q1136X were not secreted. Since these latter two mutants lack the last N-glycosylation site (N1162), the potential role of this glycosylation site for ACE secretion was studied by mutating the Asn residue of WT ACE (N1162Q ACE). The corresponding mutant was expressed in CHO cells and secreted into the culture medium similarly to WT ACE (unpublished data), showing that the N1162 glycosylation site is dispensable for ACE secretion.

In brief, after trafficking into the Golgi, WT ACE exited rapidly to reach the plasma membrane from which it was eventually secreted. The R1180P mutant followed the same ic trafficking route but it was delayed in the second glycosylation step (CNX interaction) and partially retained in the ER. It was detectable, although only to a moderate extent, at the plasma membrane. R762X, like WT ACE, was not present in the ER and transited rapidly from the Golgi to exit the cell as it lacks a membrane anchor. It was found in the cell culture medium. All other mutants were more or less retained within the ER and present in the Golgi. The missense mutants were not found at the cell surface, which was suggestive of their misfolding and ic degradation.

### Enzymatic activity of ACE mutants

ACE activity was tested, both in cell lysates and in cell culture medium, using substrates that were only moderately domain selective, i.e. HHL (Hip-His-Leu) and AcSDAcKP (Acetyl-Serine-Aspartyl-Acetyl-Lysine-Proline), a C- and an N-domain substrate, respectively. Direct ACE measurement was performed by Enzyme Linked ImmunoSorbent Assay (ELISA). Table 2 shows the presence of an ACE N-domain activity of the R828H mutant within the cell lysate comparable with WT ACE. This mutant was devoid of any C-domain activity. The mutant W594R had an overall low enzymatic activity with a preservation of the C-domain specificity and a loss of the N-domain activity. The other mutants R1180P, G1145AX and Q1136X exhibited only a residual N- and C-domain activity in the cell lysate. The R1180P mutant showed N- and C-domain activity in the cell culture medium. As expected, the R762X mutant, which consists of only the N-domain of ACE, showed an enzymatic activity specific for this domain.

**Table 2.** Enzymatic activity of WT ACE and RTD mutants

Genotype	WT	Missense mutants			Truncated mutants		
		R1180P	R828H	W594R	R762X	Q1136X	G1145AX
Cell lysates							
C-domain^a^	28.74 ± 5.08	1.57 ± 0.33	1.46 ± 0.81	5.93 ± 1.65	0.24 ± 1.65	1.62 ± 1.02	1.59 ± 0.55
N-domain^b^	15.43 ± 2.69	4.95 ± 1.34	14.46 ± 2.35	0.16 ± 0.06	2.78 ± 0.84	3.07 ± 1.88	3.25 ± 1.08
Medium							
C-domain^a^	7.35 ± 0.71	3.73 ± 0.64	UND	UND	0.36 ± 0.09	UND	UND
N-domain^b^	5.53 ± 0.52	2.99 ± 0.80	UND	UND	8.79 ± 4.08	UND	UND

ACE amount was quantified with a human ACE DuoSet Enzyme Linked ImmunoSorbent Assay. ACE activity was determined using HHL and AcSDAcKP as specific substrates for the C- and N-domains, respectively. The rate of hydrolysis of the two substrates was quantified by HPLC to obtain pmole/ng/min of the reaction products AH^a^or AcKP^b^. Data are means of three independent experiments. UND, not detectable.

### Effect of the mutation on the shedding rate of the R1180P ACE mutant

**Figure 6.**
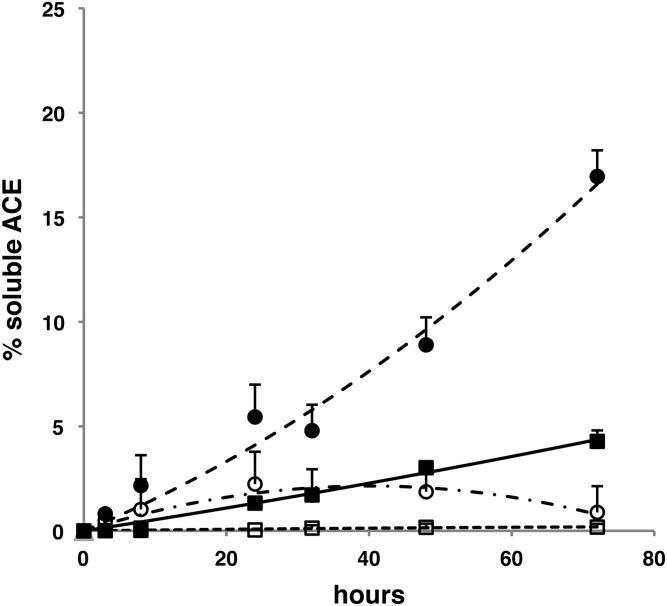
Shedding of WT ACE and of R1180P mutant in CHO cells. WT ACE (black square) and R1180P mutant (black circle) secretion in CHO cells was quantified with a human ACE ELISA in cell lysates and culture media. ACE is expressed as a percentage of the total amount of ACE in cell lysates and in culture medium. The effect of the sheddase inhibitor TAPI-1 on the solubilization rate of WT ACE (open squares) and the R1180P mutant (open circles) is indicated. Results shown are means of four points corresponding to two separate experiments.

The R1180P mutation is located 15 amino acids upstream to the beginning of the stalk region. This mutant was found to be inserted in the plasma membrane and its solubilization rate in CHO cells was markedly increased when compared with WT ACE (Fig. 6). The shedding of WT ACE and of the R1180P mutant was inhibited by TAPI-1, an inhibitor of the shedding of several cell surface proteins.

## DISCUSSION

The RAS is expressed early during human kidney development. Human ACE is detected with the other components of the system as early as 30–35 days of gestation (26–28). The importance of the RAS in embryonic renal function is shown by the dramatic consequences of ACE inhibitor treatment during pregnancy. Congenital malformations have been reported in infants with fetal exposure during the first trimester alone (29) but the fetal effects of ACE inhibitors appear to be more marked during late pregnancy and are attributed to severe hypotension and renal hypoperfusion (30,31). The role of the RAS in human kidney development has been further documented by the reports of genetic RTD due to gene inactivation of any one of the major components of the RAS (18). Interruption of RAS function leads to severe renal hypoperfusion, oligohydramnios, refractory hypotension, anuria and RTD. Among the 33 distinct ACE mutations reported recently by Gribouval *et al*. (23), several of them could be easily predicted to lead to the lack of a functional RAS and then to RTD as they induced a premature ACE truncation or a lack of correct ACE mRNA splicing. The present study is focused on the role of tissue ACE versus circulating ACE, as two mutants showed either a total lack (R762X) or a partial defect in cellular ACE insertion (R1180P). These mutants help to decipher the role of ic (tissue-bound ACE) versus secreted (circulating) ACE. Four other ACE mutants (W594R, R828H, Q1136 and G1145AX) were also studied as they provide important information about structure–activity relationship, cellular trafficking and ACE enzymatic activity. These four mutants were unable to reach the plasma membrane and were not secreted. Figure 1 summarizes the actual cellular localization of the mutants studied.

### Misfolding of the four ACE mutants W594R, R828H, Q1136X and G1145AX leads to RTD

ACE is a heavily glycosylated protein (nine glycosylation sites in the N-domain and six in the C-domain) (32,33). Proteins that are destined for the cell surface or secretion are translated in the rough ER and exported to the Golgi where additional maturation takes place. In the ER, proteins undergo primary N-glycosylation and are then transported to the Golgi complex where further glycosylation occurs. Protein glycosylation is crucial for promoting proper protein folding, ic trafficking, cell surface expression and secretion (34,35). Here, we show that WT ACE is N-glycosylated and then transported to the Golgi where it is fully glycosylated. This final modification is important for its translocation to the cell surface and for its subsequent secretion. In contrast, the ACE mutants W594R, R828H, Q1136X and G1145AX were incompletely glycosylated. They lacked complex glycan addition which accounts for their abnormal cellular trafficking and ic localization, indicative of misfolding. These four mutants had an abnormal ic distribution, being retained in the ER and associated with CNX for a longer period of time than WT ACE. To our knowledge, this is the first evidence of an extended interaction between CNX and ACE mutants.

**Figure 7.**
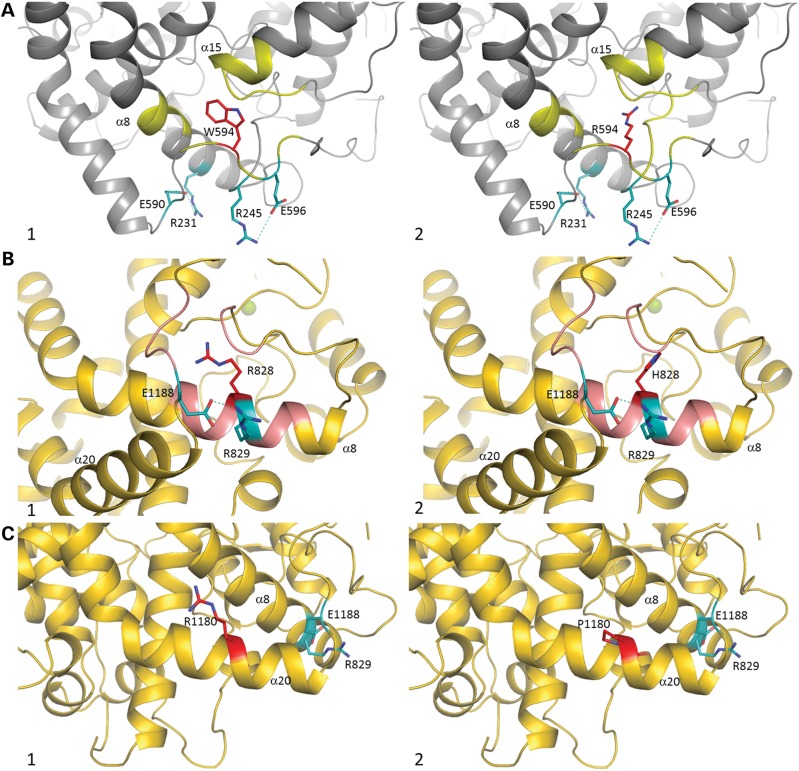
(**A**) Crystal structure of N-domain ACE (PDB code 3NXQ) (32) in grey. Residue W594 is shown in red, with salt bridges R245–E596 and R231–E590 in cyan (bond in dash lines). Interactions with W594 are highlighted in yellow. (1) Close-up view of the W594 area. (2) Modelled W594R mutation (red) with its potential interaction (yellow). (**B** and **C**) Crystal structure of C-domain ACE (PDB code 1O8A) (12) in gold. (B) Residue R828 is in red, with salt bridge R829–E1188 in cyan (bond in dash lines). Interactions with R828 highlighted in pink. (1) Close-up view of the R828 area. (2) Modelled R828H mutation (red) with its potential interaction (pink). (C) Residue R1180 in red, with salt bridge R829–E1188 in cyan (bond in dash lines). Interactions with R1180 are highlighted in pink. (1) Close-up view of the R1180 area. (2) Modelled R828H mutation (red). Secondary structure numbering is based on C-domain alone (12).

The W594R mutation is located only eight amino acids upstream to the beginning of the inter-domain bridge region. This mutant exhibited a weak C-domain catalytic activity. It is likely that the bridge region would provide enough flexibility for domain cooperativity (inter-domain interaction/s) rather than contributing to the activity of the protein. The 3D structure of the ACE N-domain (11) shows the presence of two salt bridges, between E596 and R245 and between R231 and E590, important for the stability of the C-terminal region of this domain. The W594R mutation probably disturbs the surrounding loops, normally stabilized by W594, via hydrophobic interaction with helix α8 and hydrogen bonds with helix α15. Substitution to a long polar Arg side chain may result in new hydrogen bond-mediated interactions with the side chain of H226 on helix α8 or with the main chain of T468 and G472 on helix α15 and is likely to cause the movement of the flexible loop holding W594R, as well as E590 and E596. Thus, the flexibility of the loop due to the Arg substitution would enhance its solvent accessibility and may in turn disrupt the salt bridges and hence might have an effect on the overall folding of the protein (Fig. 7A, 1 and 2). Alternatively, the proximity of the mutation to the bridge area may alter the inter-domain flexibility and affect the ACE activity, which might explain the low N-domain activity.

Based on a careful observation of the structure of the C-domain of ACE (12), we predict that the R828H mutation may induce multiple new interactions with residues of the 840 loop region which could contribute to the misfolding of the protein by the disruption of the helical (α8) structure and the salt bridge (R829–E1188) stabilizing the C-terminal loop (Fig. 7B, 1 and 2). This is in agreement with the results from enzymatic assays demonstrating the loss of C-domain activity while retaining full N-domain enzymatic potency.

The two truncated C-domain mutants Q1136X and G1145AX were not secreted. These mutants lack the last glycosylation site (N1162), the role of which was assessed by creating an ACE mutant devoid of this very glycosylation site (ACE N1162Q). The solubilization and the cellular trafficking of ACE N1162Q were not affected by the lack of glycosylation at this site, showing that this glycosylation site was dispensable for normal trafficking and sorting. Nesterovitch *et al*. (16) reported the solubilization and the presence in the blood of a truncated mutant (W1197X). The mutation is also located in the stalk region but downstream to the Q1136 and G1145 mutations. It is likely that the deletion of several secondary structural elements (helices α18-α20) is detrimental to the protein and likely to cause misfolding of truncated proteins Q1136X and G1145X. This would also result in the loss of structurally important intra-molecular interactions, such as the R829–E1188 salt bridge. Moreover, the deleted portion is in close proximity to the catalytic pocket of C-domain ACE, composed of helices α14 and α17, which are involved in zinc and chloride ion coordination, both necessary for the catalytic activity of the molecule and thus would explain the almost complete loss of C-domain activity (Supplementary Material, Fig. S2).

### R1180P mutation leads to a partially membrane-bound ACE but is compatible with life

The patient with the R1180P mutation presented transient renal insufficiency at birth and then spontaneously recovered. Clinical and biological investigation of this patient at the age of 10 years and of his two parents revealed in the patient a marked PRA increase and a marked decrease in plasma angiotensinogen concentration. This decrease in plasma angiotensinogen is likely due to an intense stimulation of the RAS in order to maintain a near to the normal Ang II level. Both the patient and his mother carried the R1180P mutation. The presence of a normal level of ACE activity in the plasma of the patient and his mother showed that R1180P is secreted and circulates in plasma *in vivo*. This is in good accordance with the *in vitro* data which showed that this mutant was bound to CHO and HEK cells and efficiently secreted into the cell culture medium. The absence of RTD phenotype in the patient's mother is explained by the presence of an ACE functional allele. The patient's father had a lower plasma ACE activity which is related to the presence of a single functional allele.

Analysis of the structural implications of the mutation (Fig. 7C, 1 and 2) would suggest a possible disruption of the C-terminal helix α20, resulting in a change of the secondary structure that would favour ACE shedding from its membrane-bound anchor. Insertion of a proline residue within this helix may cause disruption or a distortion (‘kink’) that might result in conformational alterations within the C-terminal loops and might create space for cell-surface protease entry. Although both domains conserved their enzymatic activities, they were significantly reduced compared with that of WT (Table 2). Although a mutation at position 1180 is unlikely to directly affect the N-domain due to the relative positioning of the two domains (Fig. 1), it is possible that a modification of the C-terminus of ACE could alter the inter-domain cooperativity. Further structural studies would be required to delineate this mechanism, but several previous studies have highlighted the negative cooperativity between the two domains of various ACE homologues, including human ACE (36).

### R762x mutation leads to an *in vitro* functional and secreted ACE but is deleterious *in vivo* as it results in RTD

This mutation is located in the C-domain of ACE, 197 amino acids upstream to the catalytic site. The expressed mutant was metabolically stable, appeared to be correctly folded and was very rapidly secreted into the cell culture medium. Its enzymatic activity was that expected from an ACE molecule harbouring a single N-domain. Therefore, this mutant behaved apparently as a secreted single functional N-domain ACE, without plasma membrane and tissue insertion. It is unlikely that the lethality resulting from this mutation is due to the lack of C-domain activity in terms of Ang II production. Indeed, N-domain ACE is able to hydrolyse Ang I and BK with a catalytic activity similar to that of the ACE C-domain (2,3). It is most likely that the absence of renal tissue-bound ACE is responsible for the lethality. Tissue ACE appears to be crucial for assuming correct renal perfusion, kidney development and function in humans. A somewhat similar ACE N-domain mutant has been generated in the mouse by genetic engineering by Esther *et al*. (37). The genetically modified mice had a single N-domain ACE, no tissue-bound ACE and were viable. They had a low blood pressure, renal vascular thickening and a urine concentration defect. It was concluded from this study that tissue-bound ACE was essential for the control of blood pressure and the structure and function of the mouse kidney. The difference between mice and humans in the severity of the inactivation of genes of the RAS might be due to several factors, i.e. the difference in the chronology of kidney maturation which occurs after birth in mice and which is completed during gestation in humans, and the earlier peak of RAS activity during fetal life in the human kidney, as put forward by Gubler and Antignac (38).

It is interesting to compare the *in vitro* and *in vivo* consequences of the R762X and R1180P mutations. Neither of these mutations significantly affect ACE catalytic activity but they markedly differ in the presence or the absence of cell-bound ACE. The R762X mutant is not inserted in the plasma membrane and results in perinatal cell death. The R1180P mutant is detected, albeit to a low extent, at the plasma membrane and is rapidly solubilized and secreted into the culture medium. *In vivo*, the mutant is also secreted and measurable in the patient's plasma. One hypothesis to explain the presence of anuria at birth followed by the spontaneous recovery of renal function is that the small amount of tissue R1180P ACE was sufficient to permit the restoration of a normal renal blood flow. This was most likely dependent on an intense stimulation of the RAS, which was still present 10 years post-natally and compensates the relatively low presence of tissue-bound ACE in this patient. An alternative hypothesis is that the patient has developed other pathways for Ang II and angiotensin peptide production (39,40) which may have contributed to renal function maturation. This may be the case of mast cell chymase, an enzyme able to generate Ang II from Ang I, and which is involved in human kidney disease progression (41). In any case, it is highly interesting that the patient could progressively manage a return to normal renal function, whereas the R762X mutant, totally deficient in tissue ACE could not, highlighting the importance of tissue bound ACE during fetal and perinatal life for the maturation of the human kidney.

## MATERIALS AND METHODS

### Plasmid constructs

The WT human ACE cDNA (42) was subcloned into pcDNA3.1/Zeo plasmid (Invitrogen). The plasmid constructs for R1180P, R828H, W594R, R762X, Q1136X and G1145AfsX12 (G1145A) mutants, as all other plasmid constructs, were performed by Genosphere. Mutation numbering in mature somatic ACE protein (without signal peptide) was performed according to Soubrier *et al*. (43).

### Analysis of the RAS in human plasma

Angiotensinogen and PRA were measured in family DYS66. Angiotensinogen was measured by the generation of Ang I after full hydrolysis by an excess of recombinant renin at pH 5.7, as described previously (24,44) and expressed as ng Ang I/ml. PRA is defined as renin-dependent conversion of angiotensinogen to Ang I, measured as generation of Ang I in ng/ml/h; Ang I was measured by radioimmunoassay at pH 5.7 (44). ACE activity was measured using Ang I as a substrate. After addition of Ang I, the generated Ang II was quantified by an Ab-trapping method (25).

### Antibodies and reagents

The primary Abs used in this study include a polyclonal rabbit anti-ACE Y1 against pure human kidney ACE (45), a polyclonal sheep anti-ACE HKCE (15) and commercial Abs: anti-CNX Ab from Sigma, anti- PDI mAb, anti-giantin mAb, a Golgi marker from Abcam. TAPI-1 was purchased from Calbiochem.

### Cell cultures and transfection

Established CHO-K1 and HEK293 cells were obtained from American Type Culture Collection and grown in their respective complete media. Cells were stably or transiently transfected with either WT ACE or the mutants, using Lipofectamine 2000 (Invitrogen). For stable expression in CHO cells, transfected cells were selected for resistance to zeocin and clones screened by immunoblotting.

### Proteins extract and immunoblots

Stably transfected cells were grown to confluency in complete medium, then depleted in serum-free medium Opti-MEM. The conditioned media were concentrated by centrifugation (ultrafree-4biomax-10 kDa membrane, Millipore). Cells were harvested by scraping into phosphate buffer saline (PBS) and resuspended in PBS with (3-[(3-cholamidopropyl)dimethylammonio]-1-propanesulfonate) 8 mM in the presence of a protease inhibitor cocktail (Complete, EDTA-free, Roche Diagnostics) and then centrifuged at 20,000*g* for 20 min at 4°C to remove the insoluble material. Proteins in culture media and cell lysates were loaded onto 7.5% SDS–PAGE gels for immuno-blot (IB) analysis. The membrane was probed with the Y1 Ab, revealed with the alkaline phosphatase system using AttoPhos (Promega) as a substrate. The bands intensities were quantified using Quantity One software (BioRad).

### Immunoprecipitations

Protein extracts were prepared as described previously (22) and were incubated overnight at 4°C with 3 µl of HKCE Ab and protein G-Sepharose. For co-IP with CNX, cells lysates were incubated with the HKCE Ab (3 µl) or the CNX Ab (3 µl) and immunoprecipitated proteins were visualized by IB with the Y1 Ab (IB ACE) or the CNX Ab (IB CNX).

### Pulse-chase analysis

Proteins were radiolabelled for 0.5 h as described previously (22) and immunoprecipitated as described above. Proteins were revealed by autoradiography.

### Deglycosylation

For the deglycosylation of ACE molecules, cell lysates and media were immunoprecipitated as described above. The immune complex with protein G-Sepharose was suspended in 25 µl of 1% SDS and heated at 100°C for 10 min. Supernatants were divided into three aliquots for treatment with PNGase F, Endo H or no treatment for 1 h at 37°C according to the manufacturer (New England BioLabs). Digestion products were analysed by IB with Y1 Ab.

### Cell surface localization

CHO cell lines seeded in six-well plates were grown to confluency. Cells were quickly washed with PBS and fixed with 0.4% paraformaldehyde (PFA). After washing, 3 µl of HKCE Ab in 1 ml of PBS/0.1% bovine serum albumin (BSA) were added to the cell cultures at RT. After 1 h of incubation with gentle agitation, the cells were washed three times with PBS, lysed in lysis buffer as described above and adsorbed on protein G-Sepharose (50 µl) at 4°C overnight. In another well, cells were fixed, incubated with 0.1% BSA in PBS, then lysed and incubated with HKCE Ab and protein G-Sepharose to obtain the total amount of ACE. Proteins were analysed by IB with Y1 Ab.

### IF microscopy

HEK cells were seeded on 14 mm diameter coverslips and transfected during 4 h as described above and incubated in Dulbecco's Modified Eagle Medium. 6.5, 24 or 48 h after transfection, the cells were fixed, permeabilized and labelled as previously described (22) with one of the following primary Abs: Y1 or HKCE Ab (1:3000 dilution) or with anti-PDI Ab (1:1000 dilution), anti-CNX Ab (1:2000 dilution) in PBS, 0.1% BSA and with the appropriate dye-labelled secondary Abs (Molecular Probes): anti-rabbit, anti-sheep or anti-mouse Alexa 488 or 555. For cell surface expression, cells were fixed with PFA, saturated and incubated with Y1 Ab followed by the appropriate secondary Ab. Cells were then permeabilized, saturated and incubated with the other primary Ab, HKCE and the corresponding secondary Ab.

Cells were examined with a Spinning Disk Roper (inverted Nikon Eclipse Ti microscope—head of scan Yokogawa) equipped with 405/488/561 diode lasers. Images (1392 × 1040 pixels) were obtained with a Coolsnape HQ2 camera at ×60 magnification with Metamorph (Molecular devices) software.

### ACE quantification and enzymatic activity

ACE amount in cell lysates and medium were quantified with the human ACE DuoSet ELISA (R&D systems). Shedding-kinetics studies were carried out as described above, in the presence or not of 10 µm TAPI-1 and the ACE amount was quantified by ELISA.

ACE enzymatic activity was assayed using two substrates: HHL (5 mm), as an ACE C-domain-specific substrate and AcSDAcKP (2.5 mm), as an N-domain-specific substrate. Assays were performed at 37°C for 120 min with an ACE amount quantified by ELISA to obtain ∼5% of rate hydrolysis. The rate of hydrolysis was determined and quantified using reverse-phase high-performance liquid chromatography as described previously (46). The specificity of the reaction was assessed in the presence of 10 μm Lisinopril.

### Structural models

Structural models were prepared using the X-ray crystal structures of the C-domain (PDB code 1O8A) (12) and N-domain (PDB code 3NXQ) (31) of ACE. Point mutations were introduced in Swiss-PdbViewer (47) followed by energy minimization with the GROMOS96 implementation (48). Figures were prepared with PyMOL (The PyMOL Molecular Graphics System, Version 1.5.0.4 Schrödinger, LLC).

Studies were done in accordance with the recommendations of French Ethical Committees.

## SUPPLEMENTARY MATERIAL

Supplementary Material is available at *HMG* online.

## FUNDING

This work was supported in part by Naturalia et Biologica Association (NEB) and the Foundation Bettencourt Schueller. K.R.A. wishes to thank the Medical Research Council (UK) for the project grant support (G1001685) for research on ACE. Funding to pay the Open Access publication charges for this article was provided by Naturalia et Biologica Association, College de France.

## Supplementary Material

Supplementary Data
