# Supplementary material for: Absence of cell surface expression of human ACE leads to perinatal death
Source: Hum Mol Genet. 2013 Oct 24;23(6):1479–91. doi: 10.1093/hmg/ddt535 (PMC3929087; doi:10.1093/hmg/ddt535)
Supplement: Supplementary Data [file supp_23_6_1479__index.html]

Absence of cell surface expression of human ACE leads to perinatal death — Absence of cell surface expression of human ACE leads to perinatal death — Supplementary Data 

# Absence of cell surface expression of human ACE leads to perinatal death

## Supplementary Data

Supplementary Data

**Files in this Data Supplement:**

- Supplementary Data - Doc file
