# Supplementary material for: Absence of cell surface expression of human ACE leads to perinatal death
Source: Hum Mol Genet. 2013 Oct 24;23(6):1479–91. doi: 10.1093/hmg/ddt535 (PMC3929087; doi:10.1093/hmg/ddt535)

**SUPPLEMENTARY DATA**

**Supplemental Figure S1**. Co-localization of WT ACE and RTD mutants with Golgi in HEK cells. HEK cells expressing transiently WT ACE and mutants were fixed 6h30 and 48h after transfection, permeabilized and stained with the anti-ACE Ab HKCE (green) and the Giantin Ab (red). Only merged images are shown. Scale bar 10µm.


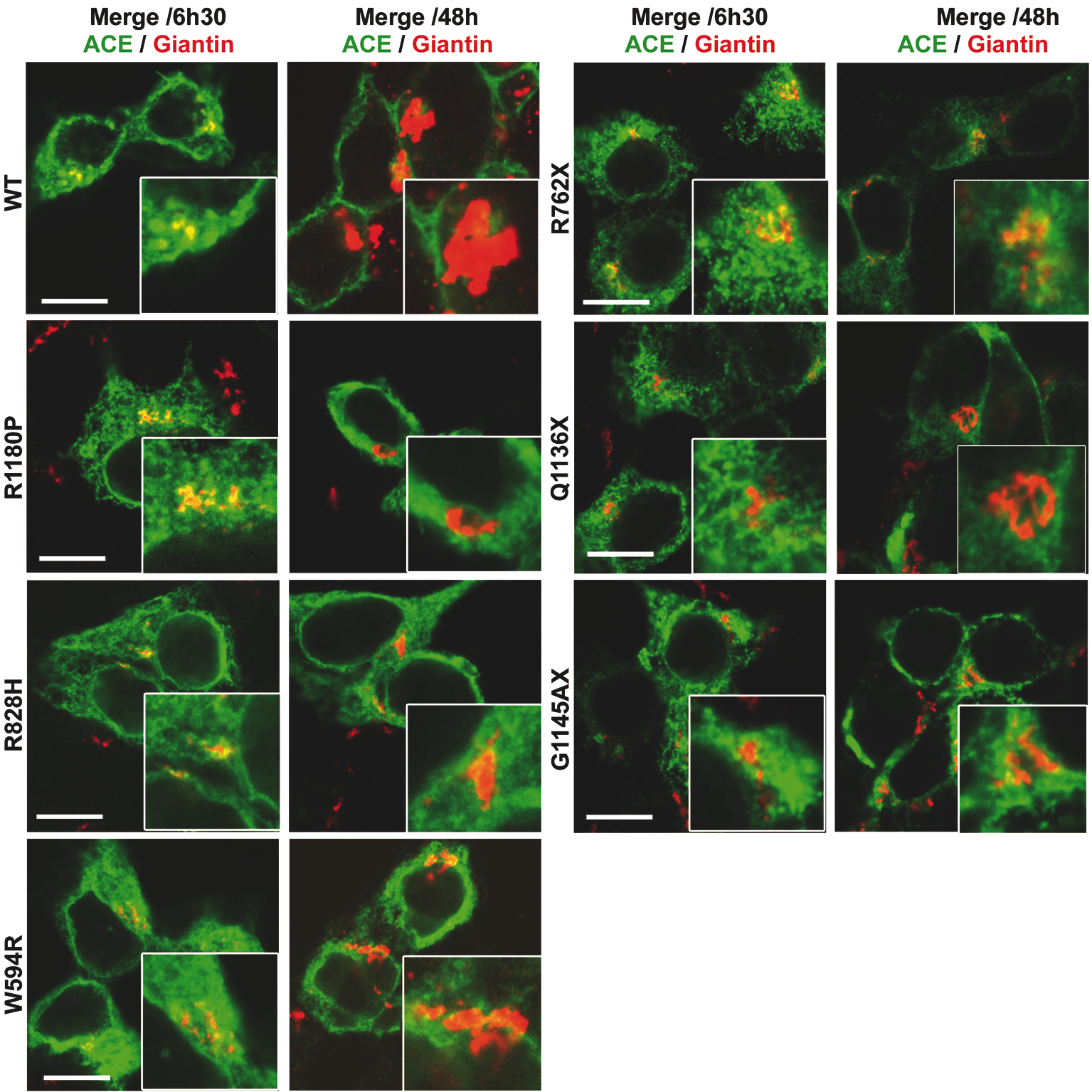


**Supplemental Figure S2.** Crystal structure of C-domain ACE (PDB Code 1O8A. Natesh et al., 2003) in gold. Location of truncated residues (for the truncated mutants described in this report) from G1145, and Q1136 are shown in red and pink colours respectively. Zinc and chloride ions shown as grey and green spheres respectively. Secondary structure numbering is based on C-domain alone (Natesh et al., 2003).


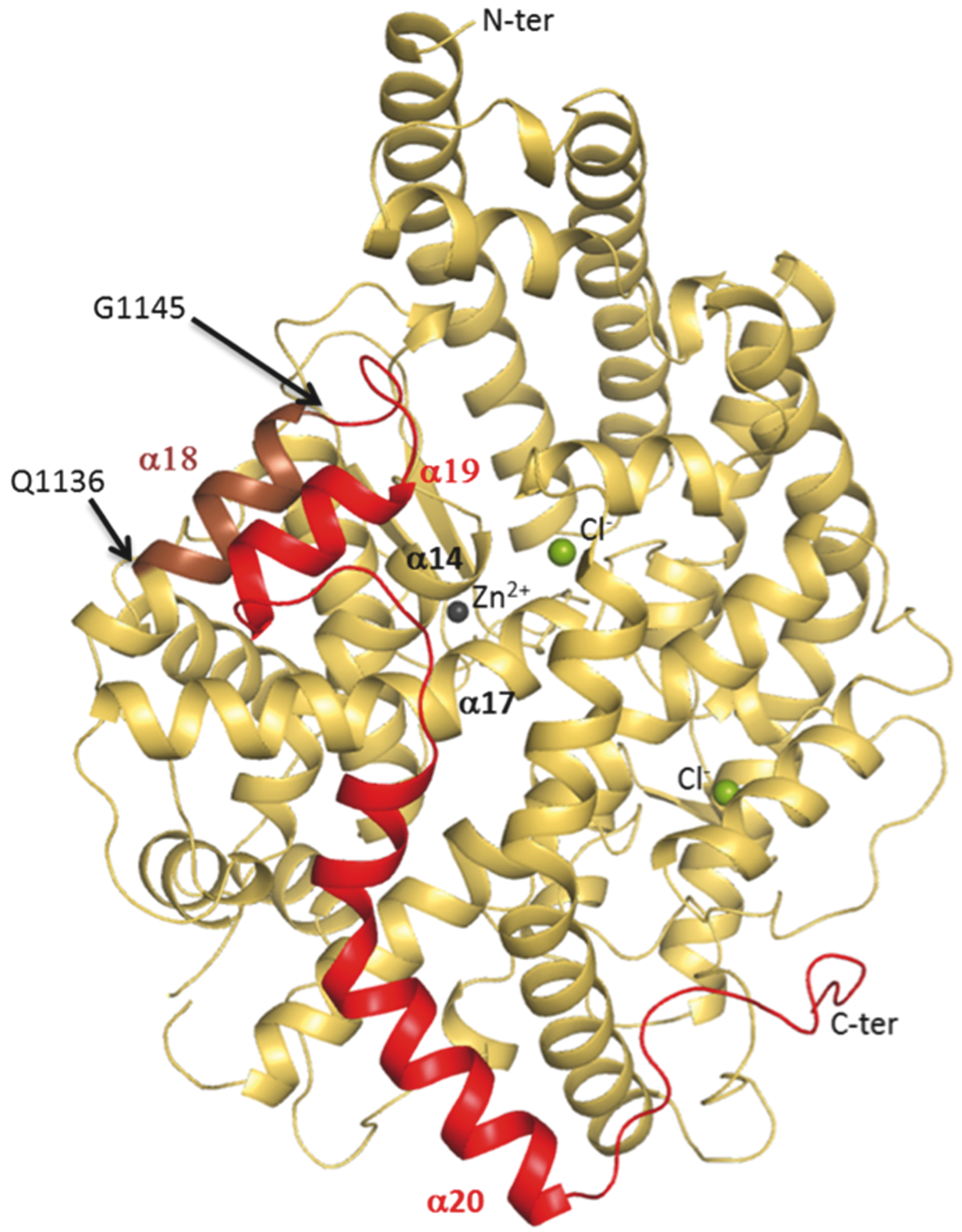

Supplement: Supplementary Data [file supp_ddt535_ddt535supp.doc]
